# Outcome and prognostic factors in patients undergoing salvage therapy for recurrent esophagogastric cancer after multimodal treatment

**DOI:** 10.1007/s00432-022-04016-y

**Published:** 2022-04-19

**Authors:** Leonidas Apostolidis, Kristin Lang, Leila Sisic, Elena Busch, Aysel Ahadova, Ramona Wullenkord, Henrik Nienhüser, Adrian Billeter, Beat Müller-Stich, Matthias Kloor, Dirk Jaeger, Georg Martin Haag

**Affiliations:** 1 National Center for Tumor Diseases (NCT) Heidelberg, Department of Medical Oncology, Heidelberg University Hospital, Im Neuenheimer Feld, 460, 69120 Heidelberg, Germany; 2 Department of Radiation Oncology, Heidelberg University Hospital, Heidelberg, Germany; 3 Department of General, Visceral and Transplantation Surgery, Heidelberg University Hospital, Heidelberg, Germany; 4 Department of Applied Tumor Biology, University Hospital Heidelberg, Clinical Cooperation Unit Applied Tumor Biology, German Cancer Research Centre (DKFZ), Heidelberg, Germany; 5 Clinical Cooperation Unit Applied Tumor-Immunity, German Cancer Research Center (DKFZ), Heidelberg, Germany

**Keywords:** Esophagogastric cancer, Perioperative treatment, Relapse, MSI, Salvage treatment

## Abstract

**Purpose:**

Perioperative systemic treatment has significantly improved the outcome in locally advanced esophagogastric cancer. However, still the majority of patients relapse and die. Data on the optimal treatment after relapse are limited, and clinical and biological prognostic factors are lacking.

**Methods:**

Patients with a relapse after neoadjuvant/perioperative treatment and surgery for esophagogastric cancer were analyzed using a prospective database. Applied treatment regimens, clinical prognostic factors and biomarkers were analyzed.

**Results:**

Of 246 patients 119 relapsed. Among patients with a relapse event, those with an early relapse (< 6 months) had an inferior overall survival (OS 6.3 vs. 13.8 months, *p* < 0.001) after relapse than those with a late relapse (> 6 months). OS after relapse was longer in patients with a microsatellite-unstable (MSI) tumor. Systemic treatment was initiated in 87 patients (73% of relapsed pat.); among those OS from the start of first-line treatment was inferior in patients with an early relapse with 6.9 vs. 10.0 months (*p* = 0.037). In 27 patients (23% of relapsed pat.), local therapy (irradiation or surgical intervention) was performed due to oligometastatic relapse, resulting in a prolonged OS in comparison to patients without local therapy (median OS 35.2 months vs. 7.8 months, *p* < 0.0001). Multivariate analysis confirmed the prognostic benefit of the MSI status and a local intervention.

**Conclusion:**

Patients relapsing after multimodal treatment have a heterogeneous prognosis depending on the relapse-free interval (if systemic treatment applied), extent of metastatic disease as well as MSI status. The benefit of additional local intervention after relapse should be addressed in a randomized trial.

**Supplementary Information:**

The online version contains supplementary material available at 10.1007/s00432-022-04016-y.

## Introduction

Platinum-based perioperative systemic treatment has significantly improved the outcome in patients with locally advanced gastric and gastroesophageal junction cancer (Cunningham et al. 2006; Ychou et al. 2011). The pivotal MAGIC phase III trial has shown a survival benefit of 13% at 5-year overall survival (OS) rate with the use of perioperative ECF chemotherapy (Epirubicin, Cisplatin, 5-FU). More, recently the perioperative use of the taxane-containing FLOT regimen has shown further improvement in overall survival (OS) in the AIO FLOT4 trial, leading to a 5-year OS-rate of 45% (Al-Batran et al. 2019). However, the majority of patients will still relapse after curatively intended multimodal treatment and die due to recurrent disease.

As a consequence, a relevant number of patients starting first-line palliative treatment have previously been exposed to a perioperative chemotherapeutical regimen, often including the most active cytotoxic substances in esophagogastric cancer: platinum compounds, fluoropyrimidines and taxanes. Thus, it is questionable whether the same chemotherapeutical regimens should be used as in de-novo metastatic patients. Though the majority of clinical trials in the metastatic first-line setting permits the inclusion of patients who received a previous neoadjuvant/adjuvant therapy more than 6–12 months ago, the rate of included patients with a relapse after previous perioperative systemic treatment—if reported at all—is less than 5–10% in recent phase III trials, limiting any conclusion regarding this subgroup (Janjigian et al. 2021; Janjigian et al. 2021; Shitara et al. 2020; Shah et al. 2021). In addition, residual toxicity from previous treatment, especially in form of sensory polyneuropathy, might prohibit the use of platinum or taxane compounds in the metastatic setting.

For patients with an early relapse after perioperative FLOT, data from second-line trials, such as the *Rainbow* trial exploring the combination of paclitaxel and ramucirumab, cannot be applied neither, as patients with a previous taxane-based treatment were not included in this trial (Wilke et al. 2014). Risk factors for relapse have been analyzed in several prospective and retrospective analyses (Smyth et al. 2016; Becker et al. 2012; Davarzani et al. 2018; Davies et al. 2014), however, there is a lack of clinical data regarding the efficacy of palliative systemic treatment in relapsed patients after perioperative treatment. In clinical routine, the relapse-free time span as well as the histological and clinical response to neoadjuvant treatment might be considered when selecting a first-line treatment in a relapsed patient.

We performed a retrospective analysis of esophagogastric cancer patients having received perioperative chemotherapy at the National Center for Tumor Diseases (NCT) Heidelberg between 2014 and 2021 to explore outcome and prognostic factors in patients with a relapse after systemic treatment in the neoadjuvant or perioperative setting.

## Methods

Patients receiving systemic treatment in the neoadjuvant or perioperative setting for locally advanced esophagogastric adenocarcinoma were prospectively included in a single-center database. The analysis of clinical data was performed retrospectively from this prospective database. The major inclusion criteria were locally advanced esophagogastric adenocarcinoma cT ≥ 2 cN0 or any cT category with cN + without evidence for distant metastases (cM0) and application of a neoadjuvant systemic treatment followed by surgery with or without postoperative adjuvant treatment. The initial tumor stage as well as the tumor stage after neoadjuvant treatment were documented. In addition, histology, known biomarkers including human epidermal growth factor receptor 2 (Her-2) and microsatellite instability (MSI) status as well as histological regression according to the tumor regression grading established by Becker et al. were documented (Becker et al. 2012). Systemic treatment regimens applied in the neoadjuvant and adjuvant setting were recorded.

The end of primary therapy was defined as the last date on which a tumor-specific therapy or intervention was performed. In patients receiving adjuvant chemotherapy, this was the date of the last administration of adjuvant chemotherapy. In patients not receiving adjuvant therapy, this was the date of the surgical tumor resection.

An early relapse was defined as a relapse occurring within 6 months after the end of primary therapy. In addition, a sensitivity analysis using a different categorization of early *relapse (modified early relapse,* defined as a relapse within 6 months after surgery, irrespective of the application of adjuvant therapy) was performed.

Patients received a structured follow-up at our institution, which consisted of alternating CT and ultrasound examinations quarterly for 2 years, followed by a semiannual assessment in the third and fourth year and annual assessment in year 5. The time point as well as location of relapse, which was diagnosed either with radiological imaging or in case of unequivocal imaging results with histological confirmation, were documented. In addition, treatment modalities and systemic regimens applied in the metastatic setting were documented. Response to systemic treatment was analyzed in analogue to RECIST 1.1 criteria.

OS after relapse (*OS relapse*) was calculated from time of radiologically or histologically confirmed relapse until death or last follow-up. OS from the start of first-line treatment (*OS first-line tx*) was calculated from the start of palliative first-line treatment until death. If no event was observed (e.g. lost to follow-up), OS was censored at the day of last subject contact.

Progression-free survival from the start of first-line treatment (*PFS first-line tx*) was calculated from the start of first-line treatment until confirmed tumor progression or death. If no event was observed (e.g. lost to follow-up), PFS was censored at the time of last tumor assessment. Differences in survival amongst groups were estimated using the log-rank test. Cox regression analysis was used to analyze the impact of different variables on time-to-event data. Frequency differences were compared using the chi-squared test or Fisher’s exact test where appropriate.

All tests were two-sided and performed at the 5% level of significance using SPSS statistical package (version 27.0; SPSS Inc., Chicago, Illinois).

This retrospective study was approved by the local Ethics committee.

## Results

Between January 2014 and June 2021, 246 patients receiving multimodal treatment for esophagogastric adenocarcinoma were included. During a median follow-up of 24.2 months, a relapse was documented in 119 patients. Patient characteristics can be found in Table 1. As expected, a higher rate of lymph node-positive resected tumors (*p* < 0.001) and a higher rate of tumors with a poor histological response according to Becker´s classification (*p* < 0.001) were observed in the relapsed cohort.

**Table 1:** Patient characteristics

	Neoadjuvant cohort *n* = 246	Relapsed cohort *n* = 119
Age (median, range)	61 years (25–85)	63 years (30–85)
Primary tumor location		
Distal Esophagus (AEG I)	79 (32.1%)	34 (28.6%)
Cardia (AEG II)	58 (23.6%)	27 (22.7%)
Subcardial carcinoma (AEG III)	14 (5.7%)	12 (10.1%)
Stomach	95 (38.6%)	46 (38.7%)
Histology		
Adenocarcinoma, intestinal type	154 (62.6%)	66 (55.5%)
Adenocarcinoma, diffuse type	51 (20.7%)	26 (21.8%)
Adenocarcinoma, mixed type	36 (14.6%)	24 (20.2%)
Adenocarcinoma with a squamous component	2 (0.8%)	1 (0.8%)
Mixed neuroendocrine non-neuroendocrine neoplasm (MiNEN)	3 (1.2%)	2 (1.7%)
Grading		
Grade1	10 (4.1%)	2 (1.7%)
Grade 2	86 (35.0%)	42 (35.3%)
Grade 3	124 (50.4%)	64 (53.8%)
Missing	26 (10.6%)	11 (9.2%)
Her-2 Status		
Negative	167 (67.9%)	95 (79.8%)
Positive	26 (10.6%)	10 (8.4%)
Missing	53 (21.5%)	14 (11.8%)
MSI-Status		
MSS*	192 (78.0%)	96 (80.7%)
MSI	16 (6.5%)	8 (6.7%)
Missing	38 (15.4%)	15 (12.6%)
Neoadjuvant therapy regimen		
Platin/fluoropyrimidine − / + anthracycline-based	63 (25.6%)	38 (31.9%)
FLOT-based	180 (73.2%)	79 (66.4%)
Platinum-based with additional irradiation	3 (1.2%)	2 (1.7%)
Tumor stage after neoadjuvant therapy		
ypT0	16 (6.5%)	1 (0.8%)
ypT1	24 (9.8%)	2 (1.7%)
ypT2	37 (15.0%)	12 (10.1%)
ypT3	129 (52.4%)	76 (63.9%)
ypT4	40 (16.3%)	28 (23.5%)
ypN0	112 (45.5%)	27 (22.7%)
ypN1	43 (17.5%)	25 (21.0%)
ypN2	38 (15.4%)	24 (20.2%)
ypN3	53 (21.5%)	43 (36.1%)
R0	225 (91.5%)	99 (83.2%)
R1	15 (6.1%)	14 (11.8%)
Rx	6 (2.4%)	6 (5.0%)
Pathological complete remission (ypT0 ypN0)	13 (5.3%)	1 (0.8%)
Histological regression (Becker classification)		
1a (complete regression)	16 (6.5%)	1 (0.8%)
1b (< 10% residual tumor)	52 (21.1%)	10 (8.4%)
2 (10–50% residual tumor)	64 (26.0%)	34 (28.6%)
3 (> 50% residual tumor)	104 (42.3%)	66 (55.5%)
Missing	10 (4.1%)	8 (6.7%)
Application of adjuvant therapy		
Yes	153 (62.2%)	62 (52.1%)
No	90 (36.6%)	57 (47.9%)
Missing	3 (1.2%)	0 (0.0%)
Number of organs involved in relapse		
1		47 (39.5%)
2		43 (36.1%)
3		6 (5.0%)
4		1 (0.8%)
5		1 (0.8%)
Missing data		21 (17.6%)
Metastatic sites after relapse		
Lymph node		43 (36.1%)
Peritoneum		36 (30.3%)
Local recurrence		23 (19.3%)
Liver		22 (18.5%)
Lung		13 (10.9%)
Bone		7 (5.9%)
Other organs		24 (20.2%)

^*^Including MSI-low tumors

The most common site of relapse were distant lymph nodes (36.1%), followed by peritoneal carcinomatosis (30.3%), the median number of metastatic sites was two.

**Fig. 1:**
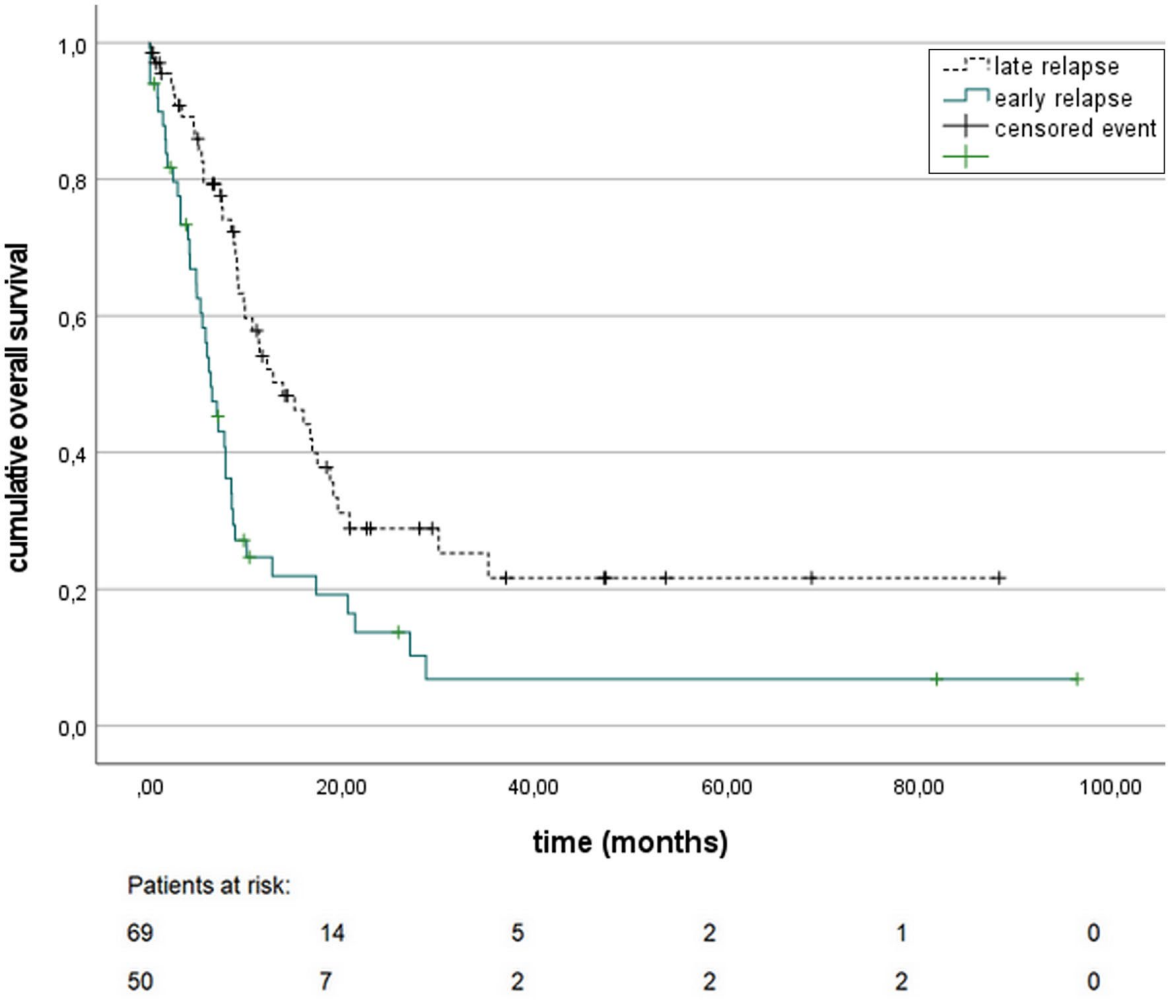
*OS relapse* in correlation to relapse-free interval

Among patients with a relapse event, *OS relapse* was 9.0 months [95% CI 7.7–10.4]; the time span between the end of the primary treatment and relapse was identified as a prognostic factor: median *OS relapse* for patients with an early relapse was significantly worse with 6.3 months [95% CI 4.9–7.8] versus 13.8 months [95% CI 8.7–18.9] in patients with a relapse more than 6 months after completion of primary treatment [Hazard Ratio (HR) 2.14 (95% CI 1.38–3.32), *p* < 0.001] (Fig. 1), whereas no significant difference was found in patients relapsing 6–12 months or after 12 months after completion of primary treatment. A superior *OS relapse* was found among patients with an MSI tumor in comparison to a microsatellite-stable (MSS) tumor [median not reached vs. 8.9 months (95% CI 7.1–10.7), HR 0.13 (95% CI 0.02–0.91), *p* = 0.039]. Among relapsed patients, the histological response according to Becker’s classification after previous neoadjuvant therapy was not associated with *OS relapse* [HR for patients with a poor histological response (Becker grade 2 or 3): 1.16 (95% CI 0.55–2.43), *p* = 0.694].

Among the 119 relapsed patients, a systemic treatment was initiated in 87 patients, with irinotecan-based regimens being the most commonly used regimens (Table 2), a rechallenge with a platinum-based doublet or triplet therapy was applied in 22 patients, checkpoint inhibitors in combination with chemotherapy or alone were applied in 10 patients, a trastuzumab-based combination was given in 5 patients. Irinotecan-based regimens were predominantly used for patients with early relapse, whereas a comparable use of platinum- or irinotecan-based therapy apart from other regimens was observed in patients with a later relapse.

**Table 2:** Treatment regimens applied as first-line therapy after relapse

Treatment regimen	Total cohort *n* = 87 (%)	Early relapse *n* = 37 (%)	Late relapse *n* = 50 (%)
Irinotecan-based regimen (+ optional FP/Ram./Tras.)	45 (51.7)	30 (81.1)	15 (30.0)
Platinum-containing doublet regimen (+ optional Tras.)	20 (23.0)	2 (5.4)	18 (36.0)
Platinum/taxane containing doublet/triplet regimen	2 (2.3)	0 (0.0)	2 (4.0)
Taxane-based regimen (+ optional Ram.)	5 (5.7)	2 (5.4)	3 (6.0)
Ctx + immune-checkpoint inhibitor (+ optional Tras.)	7 (8.0)	0 (0.0)	7 (14.0)
Immune-checkpoint inhibitor	3 (3.4)	2 (5.4)	1 (2.0)
Other	5 (5.7)	1 (2.7)	4 (8.0)

*FP* fluoropyrimidine, *Ram* ramucirumab, *Tras* trastuzumab

Overall response rate to first-line treatment was 24.1%, disease control rate was 55.1% (Table 3).

**Table 3:** Best overall response to first-line treatment

Best response	*n* = 87 (%)
Complete remission	0 (0.0)
Partial remission	21 (24.1)
Stable disease	27 (31.0)
Progressive disease	25 (28.7)
Not evaluable	14 (16.1)

*OS first-line tx* was 8.4 months [95% CI 7.5–9.4], again an inferior *OS first-line tx* was observed in early relapsed patients with 6.9 months [95% CI 5.3–8.5] vs. 10.0 months [95% CI 7.4–12.5], [HR 1.70 (95% CI 1.03–2.81), *p* = 0.037]. A comparable *OS first-line tx* was observed in patients having received irinotecan-based or other regimens (Supplement I). *PFS first-line tx* was 4.3 months [95% CI 3.6–4.9], with an inferior *PFS first-line* tx in patients relapsing within 6 months after completion of primary treatment of 3.6 months [95% CI 2.1–5.0] versus 5.3 months [95% CI 3.5–7.1] [HR 1.65 (95% CI 1.02–2.65), *p* = 0.042] in patients with a later relapse.

Analyzing outcome after relapse according to the *modified early relapse* classification showed a comparable separation in terms of *OS relapse* and *OS first-line tx*. However, when using the *modified early relapse* criteria, *PFS first-line tx* did not differ significantly any more between the two cohorts (Supplement II).

In 27 relapsed patients (23% of all relapsed patients) an oligometastatic relapse was identified that was suitable for local treatment with curative potential after interdisciplinary discussion. This approach was predominantly chosen in 24 of 69 patients with a late relapse versus only 3 of 50 patients with an early relapse (*p* < 0.001).

**Fig. 2:**
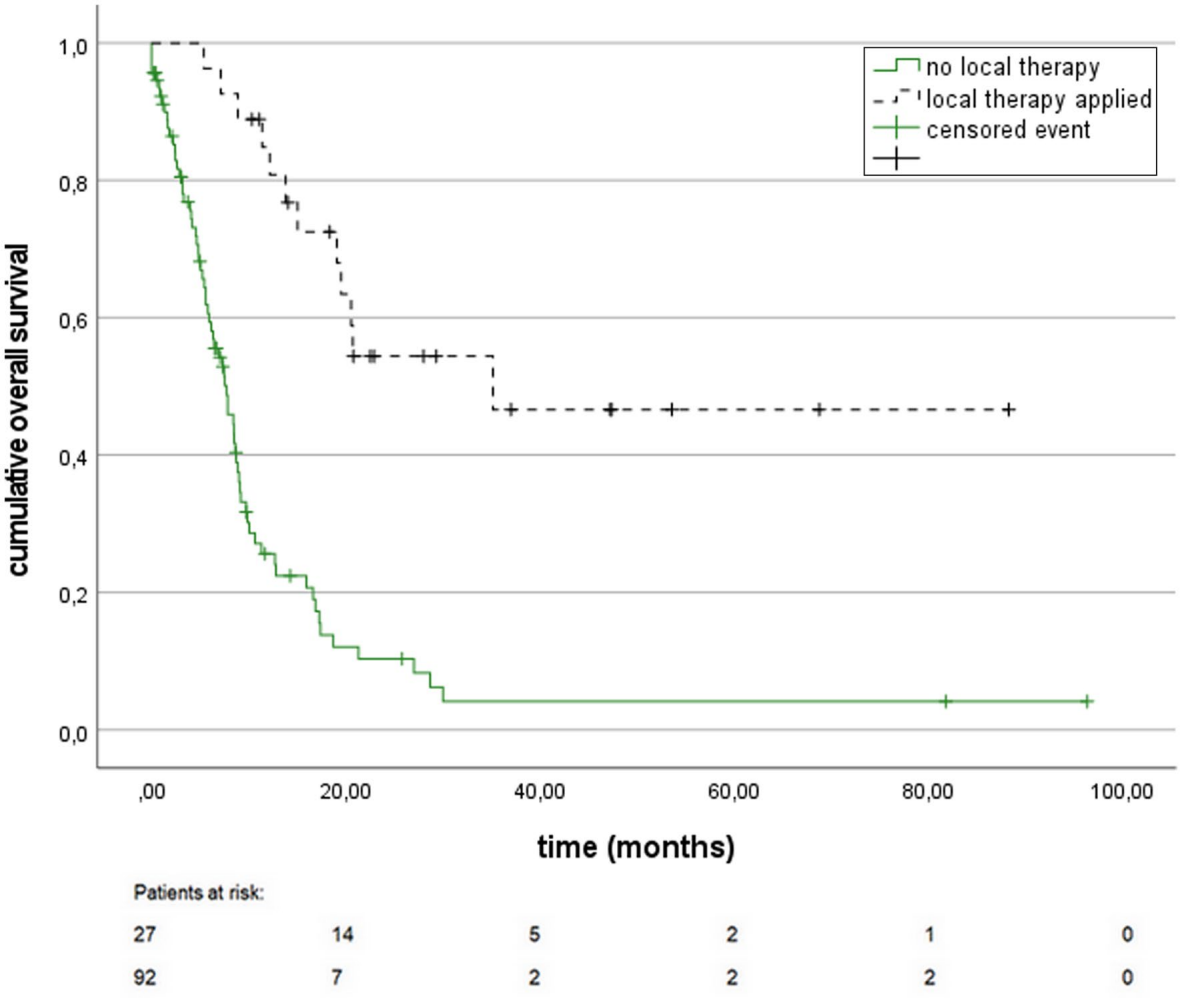
*OS relapse* in correlation to the application of a local therapy

An early local therapy, defined as a local treatment before start of systemic treatment for recurrent disease was performed in 19 patients (16.0%), with surgical resection for localized metastases or radiation of single metastatic lesion being the most commonly used treatment modalities (Table 4), in addition in 8 patients (6.7%) presenting with an oligometastatic relapse a consolidating local therapy in form of surgical resection or irradiation of single metastases was performed following application of systemic first-line therapy. Patients having received a local therapy showed a significantly improved *OS relapse* with 35.2 months [95% CI nr] vs. 7.8 months [95% CI 5.8–9.7] in patients with no curatively-intended local intervention, (HR 0.21 [95% CI 0.11–0.38], *p* < 0.0001) (Fig. 2).

**Table 4:** Local therapy applied after relapse

Early local therapy	*n* = 19 (16.0%)
Surgical resection	
Resection of brain metastasis, postoperative irradiation	3 (2.5%)
Resection of liver metastasis	2 (1.7%)
Resection of lung metastasis	2 (1.7%)
Resection of ovarian metastasis	2 (1.7%)
Resection of lymph node metastasis	2 (1.7%)
Cytoreduction/peritonectomy/HIPEC	1 (0.8%)
Resection of an isolated colonic metastasis	1 (0.8%)
Resection of adrenal metastasis	1 (0.8%)
Irradiation	
Irradiation of local recurrence	3 (2.5%)
Irradiation of lymph node metastasis	1 (0.8%)
Irradiation of liver metastasis	1 (0.8%)

Consolidating local therapy	*n* = 8 (6.7%)
Surgery	
Resection of local recurrence	1 (0.8%)
Cytoreduction/peritonectomy/HIPEC	1 (0.8%)
Irradiation	
Irradiation of liver metastasis	3 (2.5%)
Irradiation of local recurrence	2 (1.7%)
Irradiation of lymph node metastasis	1 (0.8%)

Multivariate analyses of prognostic factors for *OS relapse* including relapse-free time interval, MSI status and local therapy applied after relapse confirmed the MSI status as well as the application of local therapy as favorable prognostic markers (Table 5).

**Table 5:** Uni- and multivariate analysis of prognostic markers for overall survival after relapse

	Univariate analysis		Multivariate analysis	
	Hazard ratio	*p*	Hazard ratio	*p*
Relapse < 6Mon/ ≥ 6Mon*	2.14 [95% CI 1.38–3.32]	< 0.001	1.27 [95% CI 0.77–2.09]	0.352
MSI-tumor vs. MSS tumor*	0.13 [95% CI 0.02–0.91]	0.039	0.14 [95% CI 0.02–0.98]	0.048
Any local therapy (yes/no*)	0.21 [95% CI 0.11–0.38]	< 0.0001	0.29 [95% CI 0.14–0.59]	< 0.001
Poor histological response (Becker category 2/3 vs. 1a/1b* after neoadjuvant therapy)	1.16 [95% CI 0.55–2.43]	0.694	Not included	

^*^Reference category

## Discussion

Despite advances in the multimodal treatment of locally advanced esophagogastric cancer, more than half of patients will relapse during follow-up and die due to metastatic disease.

As a consequence, the proportion of patients presenting with metastatic disease having already received perioperative systemic treatment for previous localized disease remains high. The development of tumor clones resistant to previously applied drugs as well a residual toxicity from previous chemotherapy, complicate the selection of an appropriate treatment regimen for recurrent disease.

The present study aimed to analyze prognostic factors in patients with a relapse after multimodally treated esophagogastric cancer.

The majority of patients in our cohort had received FLOT-based perioperative treatment; for this subgroup, no data from recent phase III trials exploring second-line treatment can be applied (Wilke et al. 2014; Shitara et al. 2018), as this group of patients has already been exposed to both a platinum and a taxane compound.

In a univariate analysis *OS relapse* was clearly correlated to the time span between the end of primary therapy and the relapse occurring: patients with an early relapse, directly or within 6 months after completion of surgery with/without adjuvant chemotherapy, have a significantly worse *OS relapse* of 6.3 months, comparable to that seen with second-line chemotherapy in Caucasian patients (Wilke et al. 2014; Ford et al. 2014; Thuss-Patience et al. 2011).

Using a different definition of early recurrence (*modified early relapse*, defined as a relapse within 6 months after surgery), a similar separation in term of *OS relapse* and *OS first-line-tx* was observed. However, using this category, the prognostic value in terms of *PFS first-line* was worse, with no significant difference being observed any more. This is most likely due to the fact that patients with an early relapse after adjuvant chemotherapy have a worse outcome assuming platinum- and optional taxane-refractory tumors, even if the relapse occurs more than 6 months after surgery.

Among relapsed patients, those with an MSI tumor had a very favorable outcome, although not all of them had received checkpoint inhibition. These data are in line with several reports suggesting a beneficial outcome of stage IV MSI tumors (Haag et al. 2019; Giampieri et al. 2017; Polom et al. 2018; Busch et al. 2021). The higher immunological control caused by a higher mutational burden associated with the presentation of neoantigens might contribute to the indolent clinical course observed in this molecular subgroup.

Irinotecan-based regimens were chosen in the majority of patients as a consequence of proposed resistance against platinum- or taxane-containing therapies (especially in early-relapsed patients). Irinotecan-based regimens have shown efficacy both as palliative first-line therapy (Guimbaud et al. 2014) as well as in platinum-pretreated patients (Thuss-Patience et al. 2011; Hironaka et al. 2013). In addition, persisting sensory polyneuropathy is a common problem among patients having received FLOT-based chemotherapy perioperatively, so irinotecan-based regimens were also often selected among patients with a later relapse. The ongoing phase III *Ramiris* trial will help to define the benefit of the irinotecan-based *Folfiri* regimen and ramucirumab in taxane-pretreated patients (NCT03081143).

Recently the combination of PD-1 inhibition and platinum-based chemotherapy has shown a superior OS in patients with Her-2 negative adenocarcinoma and a PD-L1 combined prognostic score (CPS) of more than 5 or 10 respectively (Janjigian et al. 2021; Sun et al. 2021). Although it is unknown whether the addition of a PD-1 inhibitor to a platinum-based chemotherapy can overcome a resistance against platinum compounds in patients relapsing after perioperative platinum-based treatment, activity of PD-1 inhibitors both as monotherapy and in combination with chemotherapy across different lines of treatment merits further investigation in relapsed patients with a PD-L1 CPS of more than 5 or 10 respectively (Janjigian et al. 2021; Sun et al. 2021; Wainberg et al. 2021; Lei et al. 2021). Beyond PD-L1 expression and Her-2 expression, further molecularly stratified approaches are highly needed to improve outcome after failure of perioperative chemotherapy, given the modest efficacy of any cytotoxic drug in patients having failed platinum- and taxane-based treatment, such as FLOT.

A subset of our patients had a unilocular relapse that was suitable for local therapy in forms of surgical resection or irradiation after interdisciplinary consultation. Clearly, those patients would not have been identified without a structured follow-up, as clinical symptoms cannot be expected in an oligo-relapsed situation. The OS observed within this small subgroup clearly favored this approach with long-time control that could lead to sustained remission or even cure in single patients. So far, a structured follow-up has not shown to increase survival in esophagogastric cancer in a randomized setting (Bjerring et al. 2019). The plateau observed among patients having received a local therapy after relapse strongly suggest that the benefit in terms of OS is not caused by a lead-time bias, as a systemic treatment alone would probably not have been associated with the same sustained efficacy. Despite the promising overall survival, only an adequately powered randomized trial exploring a structured follow-up and interdisciplinary discussion of localized relapses in comparison to symptom-based follow-up could answer the question regarding the benefit of early identification and local treatment of relapses and thereby overcome the limitations of this retrospective analysis.

When analyzing the prognostic factors in multivariate analysis, MSI and a local intervention are confirmed as prognostic markers, however, the role of the relapse-free interval as a prognostic factor is less clear, as the correlation did not reach statistical significance. Apart from the limited number of patients in each subgroup, another possible explanation could be that, given the possible resistance to chemotherapeutical compounds, the relapse-free interval seems to be more relevant in patients receiving systemic treatment compared to those receiving local therapy, e.g. irradiation of an isolated lymph node metastasis. In addition, there is an overlap between patients with a late relapse and patients having received a local therapy in forms of radiation or surgery, as local interventions were predominantly performed in patients with a late relapse.

In summary, our study shows that patients with a relapse after multimodal therapy for esophagogastric adenocarcinoma have a heterogeneous prognosis based on clinical and molecular biomarkers. The beneficial outcome observed with local interventions in patients with an oligometastatic relapse merits further investigation in a randomized trial.

## Supplementary Information

Below is the link to the electronic supplementary material.

Supplementary file1 (DOCX 14 kb)
