# Supplementary material for: Outcome and prognostic factors in patients undergoing salvage therapy for recurrent esophagogastric cancer after multimodal treatment
Source: J Cancer Res Clin Oncol. 2022 Apr 19;149(4):1373–82. doi: 10.1007/s00432-022-04016-y (PMC10020279; doi:10.1007/s00432-022-04016-y)
Supplement: Supplementary file 1 — Supplementary file1 (DOCX 14 kb) [file 432_2022_4016_MOESM1_ESM.docx]

**Supplement I**

**Overall survival in correlation to the application of an irinotecan-based regimen**

| **All patients with first-line treatment (n=87)** | | | |
| --- | --- | --- | --- |
|  | Irinotecan-based regimen  (n=45) | Non irinotecan-based regimen*  (n=42) |  |
| **Median *OS first-line Tx*** | 8.3 months  [95% CI 7.3-9.4] | 9.0 months  [95% CI 6.4-11.6] | HR 1.11  [95% CI 0.67-1.83]  p=0.685 |
| **Early relapsed patients with first-line treatment (n=37)** | | | |
|  | Irinotecan-based regimen  (n=30) | Non irinotecan-based regimen*  (n=7) |  |
| **Median *OS first-line Tx*** | 6.5 months  [95% CI 5.0-8.1] | 7.0 months  [95% CI 2.6-11.4] | HR 0.77  [95% CI 0.29-2.05]  p=0.593 |
| **Late relapsed patients with first-line treatment (n=50)** | | | |
|  | Irinotecan-based regimen  (n=15) | Non irinotecan-based regimen*  (n=35) |  |
| **Median *OS first-line Tx*** | 10.6 months  [95% CI 7.4-13.7] | 10.0 months  [95% CI 5.1-14.9] | HR 0.91  [95% CI 0.43-1.92]  p=0.801 |

*reference category

**Supplement II**

Sensitivity analysis using a different definition of early relapse (*modified early relapse*, defined as <6 months vs. ≥6 months after primary tumor resection, irrespective of the administration of adjuvant chemotherapy).

***OS relapse, OS first-line Tx and PFD first-line Tx* in correlation to *modified early relapse***

|  | **Modified early relapse** | **Modified late relapse*** |  |
| --- | --- | --- | --- |
| **Median *OS relapse*** | 6.3 months  [95% CI 4.9-7.8] | 12.2 months  [95% CI 6.7-17.7] | HR 2.18 [95% CI 1.37-3.47], p=0.001 |
| **Median *OS first-line Tx*** | 6.5 months  [95% CI 4.7-8.3] | 9.4 months  [95% CI 7.2-11.5] | HR 1.96 [95% CI 1.14-3.38], p=0.015 |
| **Median *PFS first-line Tx*** | 3.9 months  [95% CI 2.2-5.7] | 4.6 months  [95% CI 3.3-6.0] | HR 1.62 [95% CI 0.97-2.71], p=0.067 |

*reference category

**Uni- and multivariate analysis of prognostic markers for *OS relapse***

|  | **Univariate Analysis** | | **Multivariate Analysis** | |
| --- | --- | --- | --- | --- |
|  | **Hazard ratio** | **p** | **Hazard ratio** | **p** |
| Modified early relapse <6Mon/≥6Mon* | 2.18 [95% CI 1.37-3.47] | 0.001 | 1.59 [95% CI 0.96-2.64] | 0.071 |
| MSI-Tumor vs. MSS Tumor* | 0.13 [95% CI 0.02-0.91] | 0.039 | 0.12 [95% CI 0.02-0.89] | 0.038 |
| Any local therapy  yes/no* | 0.21 [95% CI 0.11-0.38] | <0.0001 | 0.29 [95% CI 0.14-0.58] | <0.001 |
| Poor histological response  (Becker category 2/3 vs. 1a/1b* after neoadjuvant therapy) | 1.16 [95% CI 0.55-2.43] | 0.694 | not included |  |

*reference category
